# Aptamer-Dendrimer Bioconjugates for Targeted Delivery of miR-34a Expressing Plasmid and Antitumor Effects in Non-Small Cell Lung Cancer Cells

**DOI:** 10.1371/journal.pone.0139136

**Published:** 2015-09-25

**Authors:** Hongmei Wang, Xin Zhao, Caihong Guo, Dunqiang Ren, Yandong Zhao, Wei Xiao, Wenjie Jiao

**Affiliations:** 1 Department of Respiratory, The Affiliated Hospital of Qingdao University, Qingdao, P.R. China; 2 Department of Thoracic Surgery, The Affiliated Hospital of Qingdao University, Qingdao, P.R. China; 3 Department of Respiratory, Qilu Hospital, Shandong University Medical School, Jinan, P.R. China; 4 The Clinic of North Sea Fleet of PLA Navy, Qingdao, P.R. China; Universidad de Castilla-La Mancha, SPAIN

## Abstract

Metastasis and drug resistance are major barriers for the treatment of non-small cell lung cancer (NSCLC). To explore new therapeutic options, we successfully encapsulated MicroRNA-34a (miR-34a), a potent endogenous tumor suppressor in NSCLC into S6 aptamer-conjugated dendrimer to form lung cancer-targeted gene delivery nanoparticles (PAM-Ap/pMiR-34a NPs). PAM-Ap/pMiR-34a NPs had a diameter of 100–200 nm and Zeta potential of ~30 mV at applied N/P ratio. The aptamer conjugation significantly improved cellular uptake as well as gene transfection efficiency of PAM-Ap/pMiR-34a NPs in cultured NSCLC cells. We showed that PAM-Ap/pMiR-34a NPs enhanced the regulation of targeted genes, BCL-2 and p53 *in vitro*. In addition, we revealed PAM-Ap/pMiR-34a NPs significantly inhibited cell growth, migration, invasion and induced apoptosis of lung cancer cells compared with non-targeted NPs. The method provided a novel therapeutic strategy for the experimental treatment of NSCLC.

## Introduction

Lung cancer is the leading cause of cancer-related death around the world [1]. Approximately 85% of all the clinical lung cancer cases are non-small cell lung cancer (NSCLC) [2, 3]. Despite numerous advances in surgical treatment, chemotherapy and molecular targeted agents for NSCLC, metastasis and multidrug resistance are still major causes for failures in treating lung cancer patients [4, 5]. Thus, it is highly desirable to develop new therapeutic approaches beyond conventional treatment for NSCLC. MicroRNAs (miRNAs) have been shown to play an important role in the progress of human cancers and can be effective targets for cancer therapy [6]. MiRNAs are small RNAs of ~22 nucleotide long that are critical regulators of gene expression [7]. Mature miRNAs target mRNA at complementary sites, which leads to mRNA degradation and translational suppression [8, 9]. Among more than 700 human miRNAs identified, miR-34a is a transcriptional target of tumor suppressor p53 [10], and down-regulated miR-34a expression correlate with a high probability of relapse in NSCLC patients [11]. Moreover, overexpression of miR-34a inhibits the growth and metastasis of NSCLC by regulating several tumor suppressors or oncogenes, such as p53, BCL-2, c-Met and CDK4 [12]. Previous reports revealed that overexpression of miR-34a could prevent the progression and metastasis of NSCLC, as well as enhance chemotherapy sensitivity.

The development of miRNA-based therapeutics has becoming a promising alternative strategy to conventional treatment, including chemotherapy and radiotherapy for NSCLC treatment. However, the clinical application of miRNAs encounters many biological challenges, such as rapid blood clearance, poor stability in serum and inadequate cellular uptake [13]. Tumor targeting nano-delivery systems have been developed to overcome these problems [14, 15]. Gene-loaded nanocomplexes are considered to be a better fit for cancer therapy due to their passive accumulation in solid tumors by their enhanced permeability and retention (EPR) properties in the fast-growing tumors [16]. Functionalization of the polymers with specific cell-targeting ligands can enhance their tumor accumulation more than EPR effect [17], which improves transfection efficiency and biocompatibility of those gene-loaded nanocomplexes.

Polyamidoamine (PAMAM) are cationics with dendritic structure. Through electrostatic interactions, PAMAM and negatively charged pDNA form nanocomplexes which are widely used as non-viral gene vectors to transfect exogenous DNA or RNA into cells [18]. Importantly, the molecule weight and the charge density of PAMAM can be easily manipulated to have superior safety. To date, a wide variety of potential biomarkers for lung cancer have been identified, such as EGFR, TP53, CA-125 and CEA, but the absence of specificity and sensitivity was still a problem in the practice of targeted drug delivery [19].

Aptamers are short, single stranded oligonucleotides (DNA or RNA) selected from SELEX process (systematic evolution of ligands by exponential enrichment). Compared with antibodies, the advantages of aptamers are distinct, such as convenient synthesis and modification, high affinity and specificity to the target molecules, low immunogenicity, less toxicity, higher stability and rapid tissue penetration [20, 21]. Therefore, aptamer-conjugated nanocomplexes can deliver oligonucleotides in a cell-type specific manner with the improved transfection efficiency and/or reduced side effects to normal cells [22]. Several cell-specific aptamers have been used for gene delivery, but an aptamer-conjugated nanocomplex used in the treatment of NSCLC is limited. In our work, we developed PAMAM-PEG-aptamer connection (PAM-Ap) using S6, an aptamer selected against A549 lung cancer cells by cell-SELEX [23] and adopted to functionalize PAMAM G5.0, then we mixed the PAM-Ap with pMiR-34a to construct PAM-Ap/pMiR-34a nanoparticles (PAM-Ap/pMiR-34a NPs) to deliver of pDNA to lung cancer cells. Next we assessed the gene transfection efficiency and antitumor effect of PAM-Ap/pMiR-34a NPs in A549 cells. Our data show the Aptamer-dendrimer bioconjugates system is an efficient way to deliver the miR-34a expressing plasmid to non-small cell lung cancer cells.

## Materials and Methods

### Materials

Polyamidoamine (PAMAM, ethylene diamine core, G5.0) and 4',6-diamidino-2-phenylindole (DAPI) were obtained from Sigma-Aldrich (St. Louis, MO). Maleimide polyethylene glycol succinimidyl ester (Mal-PEG-NHS, Mw 2000) was purchased from Bio-matrix Inc. (Jiaxing, China). Gelred^TM^ DNA gel stain was purchased from Biotium (CA, USA). Fetal bovine serum (FBS), RPMI-1640 culture medium, Dulbecco's phosphate-buffered saline (DPBS), trypsin, penicillin-streptomycin and YOYO-1 iodide were purchased from Invitrogen/Life Technologies (Carlsbad, CA). Annexin V-FITC and propidium iodide (PI) staining kit were from BD Biosciences (San Jose, CA). Cell Counting Kit 8 (CCK-8), RIPA protein lysis buffer and BCA protein assay kit were purchased from Beyotime (Nanjing, China). A549 cell binding aptamer (S6, sequence: GTGGCCAGTCACTCAATTGGGTGTAGGGGTGGGGATTGTGGGTTG) with sulfhydryl group at the 5’-end was synthesized by RiboBio Co. Ltd. (Guangzhou, China). The miR-34a and enhanced green fluorescent protein (EGFP) co-expressing plasmid (pMiR-34a, sense: 5'-UGGCAGUGUCUUAGCUGGUUGU-3', antisense: 5'-AACCAGCUAAGACACUGCCAUU-3') and negative control plasmid (pMiR-NC, sense: 5′-UUCUCCGAACGUGUCACGUTT-3′, antisense: 5′-ACGUGACACGUUCGGAGAATT-3′) were obtained from SunBio Co.Ltd (Shanghai, China). A549 human NSCLC cell line was obtained from the Institute of Biochemistry and Cell Biology, SIBS, CAS (China). Cells were cultured in RPMI-1640 medium supplemented with 10% FBS, penicillin (100 IU/mL) and streptomycin (100 mg/mL) at 37°C incubator with 5% CO_2_.

### Synthesis of aptamer conjugated dendrimer (PAM-Ap)

To synthesize the aptamer conjugated dendrimer, 100mg PAMAM (methanol solution) was dried under nitrogen and dissolved in 5 mL PBS (pH 7.2). Then 20mg Mal-PEG-NHS (Mw 2000) was added to the PAMAM solution and stirred for 6 h, and then the mixture was dialyzed in distilled water using dialysis membrane (MWCO 3500 Da) for 24 h to remove unreacted Mal-PEG-NHS, followed by lyophilization to get pegylated PAMAM (PAM-PEG-Mal). For the conjugation of S6 aptamer, 25 mg PAM-PEG-Mal was dissolved in 2 mL PBS (pH 7.2). Then 50 nM S6 aptamer were mixed with the solution for 6 h, the product was then dialyzed against distilled water using dialysis membrane (MWCO 7500 Da) for 12 h and lyophilized to get PAMAM-PEG-Ap (PAM-Ap).

### Formulation and characterization of pDNA-loaded nanoparticles

For the pDNA binding ability evaluation, 1 μg of pDNA was mixed with PAM-Ap at N/P ratio ranging from 0.5 to 16 in PBS (pH 7.2). Each sample was vortexed for 20 s, incubated for 30 min at room temperature to form PAM-Ap/pDNA NPs. Then samples were electrophoresed in 1.5% agarose gel containing Gelred^TM^ for 20 min (100 V), and the bands of pDNA were visualized under UV light. For particle size and Zeta potential measurement, PAM-Ap/pDNA NPs were prepared at different N/P ratio in distilled water and determined by a Zetasizer Nano ZS (Malvern Inc., Westborough, MA). For the serum stability test, the naked pMiR-34a, PAM/pMiR-34a and PAM-Ap/pMiR-34a complexes (N/P = 40) were incubated in 50% FBS at 30 μg/mL. The mixtures were incubated at 37°C for different time intervals. After that, 20μL of the mixtures were treated with 10 μL heparin solution (1000 U/mL) to release the loaded pDNA following electrophoresis on a 1.0% w/v agarose gel containing Gelred^TM^ for 15 min. The undegraded pDNA were visualized under UV light.

### Flow cytometry for cellular uptake assay

A549 cells were seeded into 6-well plates at a density of 3×10^5^ cells per well and cultured for 24 h. PAM-Ap/pDNA or PAM/pDNA NPs were prepared with YOYO-1 labeled pDNA (1 molecule of YOYO-1 iodide to 20 base pairs of nucleotide) at N/P ratio of 40. Before transfection, the culture medium was removed. Then 2 mL of serum-free RPMI 1640 medium containing PAM-AP/pDNA or PAM/pDNA NPs were added to the each well (pDNA 4 μg/well). After 4 h incubation, the transfection medium was removed. The cells were washed, trypsinized and resuspended in DPBS and samples were immediately determined by flow cytometry (BD, San Jose, CA). For competition study of S6 aptamer mediated cellular uptake of PAM-Ap, A549 cells were seeded in 6-well plates, 30 min before PAM-Ap/pDNA NPs treatment, a 50-fold excess of S6 aptamer was added to the culture medium and incubated for 4 h before analyzed the cells as above.

### Confocal laser scanning microscopy (CLSM) study

A549 cells were seeded into glass coverslip in 24-well plates at a density of 5×10^4^ per well and cultured for 24 h. The YOYO-1 labeled PAM-Ap/pDNA or PAM/pDNA NPs were prepared with the same method as described above. The cells were transfected with PAM-AP/pDNA or PAM/pDNA NPs at a pDNA concentration of 1 μg/well. After 4 h of incubation, cells were washed and fixed with 4% formaldehyde, stained with DAPI. The extracellular polymers/pDNA nanoparticles were washed with DPBS containing heparin. The images were taken with confocal laser scanning microscope (Olympus, Japan).

### Transfection efficiency evaluation

To evaluate the transfection efficiency, 1.5×10^5^ of A549 cells were seeded into 12-well plates and cultured for 24 h. A549 cells were transfected with PAM-AP/pMiR-34a or PAM/pMiR-34a NPs (2 μg pMiR-34a per well) at N/P ratio of 10, 20 and 40 for 4 h. After transfection, medium was replaced and cells were cultured for another 48 h. The EGFP expressing cells were imaged by fluorescence microscope (Leica, Germany) and quantified by flow cytometry.

### RNA isolation and qPCR analysis

The levels of BCL-2 and p53 mRNA were determined by quantitative Real-Time PCR (qRT-PCR). A549 cells were seeded into 6-well plates at a density of 3×10^5^ per well and cultured for 24 h. The transfection of PAM-AP/pMiR-34a or PAM/pMiR-34a NPs was conducted as described above. After 48 h of incubation, culture medium was removed and cells were washed with PBS. Total RNA was isolated using RNeasy mini-kits (Qiagen, Germantown, MD) according to manufacturer’s protocol. BCL-2 and p53 mRNA levels were quantified using iScript^TM^ one-step RT-PCR kit with SYBR green (Bio-Rad, CA) using iQ^TM^5 RT-PCR detection system. Relative gene expression levels were calculated following a comparative Ct method against endogenous control β-actin expression level. Data are normalized to BCL-2 or p53 expression level of untreated cells. Sequences of gene-specific primers for qPCR are as following: β-actin (sense) 5’-GGATCCGACTTCGAGCAAGAGATGGCCAC-3’ (antisense) 5’-CAATGCCAGGGTACATGGTGGTG-3’; BCL-2 (sense) 5’-TTGGATCAGGGAGTTGGAAG-3’ (antisense) 5’-TGTCCCTACCAACCAGAAGG-3'; p53 (sense) 5’-ACCAGGGCAGCTACGGTTTC-3’ (antisense) 5’-CCTGGGCATCCTTGAGTTCC-3’.

### Western blot analysis

A549 cells were cultured and treated as as described above. Total protein was extracted by RIPA lysis buffer and was quantified by BCA protein assay kit. 40 mg of protein were loaded and separated on 10% Ready Gel Tris-HCl Gel (Bio-Rad, CA) and then transferred to a polyvinylidene fluoride (PVDF) membrane (Millipore, Bedford, MA). The PVDF membrane was blocked with 5% skim milk for 1 h, and probed with primary antibodies at room temperature for 1 h. Subsequently, the membrane was stained with HRP-conjugated secondary antibody and the bands were detected with the ECL Plus kit (Beyotime, Nanjing, China).

### Cytotoxicity assay

In vitro cytotoxicity was evaluated by CCK-8 assay. A549 cells were seeded into 96-well plates at a density of 6,000 cells per well and cultured overnight. The cells were transfected with PAM-Ap/pMiR-NC, PAM/pMiR-34a or PAM-Ap/pMiR-34a NPs for 4 h (2 μg/mL pMiR-34a), then the medium was replaced and cells were further incubated for 24 h, 48 h, 72 h or 96 h. After removing the media, 100 μL of culture medium containing 10 μL CCK-8 was added into each well and incubated for another 1 h, the absorbance of each well was measured using a microplate reader (Thermo Scientific, MUTISKAN MK3) at 450 nm wavelength. The cell viability of A549 cells was expressed relative to untreated cells.

### Cell apoptosis analysis

The A549 cell apoptosis were determined by flow cytometry as reported previously [24]. Briefly, A549 cells were seeded in 12-well plates at a density of 1×10^5^ cells per well and incubated overnight. The cells were transfected with PAM-Ap/pMiR-NC, PAM/pMiR-34a or PAM-AP/pMiR-34a NPs for 4 h (2 μg/mL pDNA), then the transfection medium was replaced and cells were further incubated for 48 h. Then cells were washed, trypsinized and collected by centrifugation. The cell apoptosis was assessed by flow cytometry after staining with Annexin V-FITC and propidium iodide (PI) staining kit according to manufacturer’s protocol.

### *In vitro* migration and invasion assay

The migration and invasion ability of A549 cells was evaluated using the modified 6.5 mm transwell chamber with polycarbonate membranes (8.0-mm pore size) (Costar, Cambridge, Mass). A549 cells (1×10^5^ cells) were transfected for 24 h and were trypsinized and suspended in 200 μL of serum free RPMI 1640 medium. Then the cells were seeded on the top chambers with the uncoated or precoated with 20 mg of Matrigel (for migration and invasion assay, respectively). Culture medium containing 10% FBS in the bottom chamber was used as the chemoattractant. After 24 h of incubation, non-migrated or non-invading cells were wiped off with cotton buds from the top chamber. Cells on the underside of the chamber were fixed with 4% paraformaldehyde, then stained with 0.5% crystal violet solution for 1 h and counted under a microscope in five predetermined fields.

### Statistical analysis

Data were shown as the mean ± SD. One-way analyses of variance (ANOVA) were conducted. P-value < 0.05 was defined as statistically significant.

## Results and Discussion

### Synthesis and characterization of the materials

**Fig 1:**
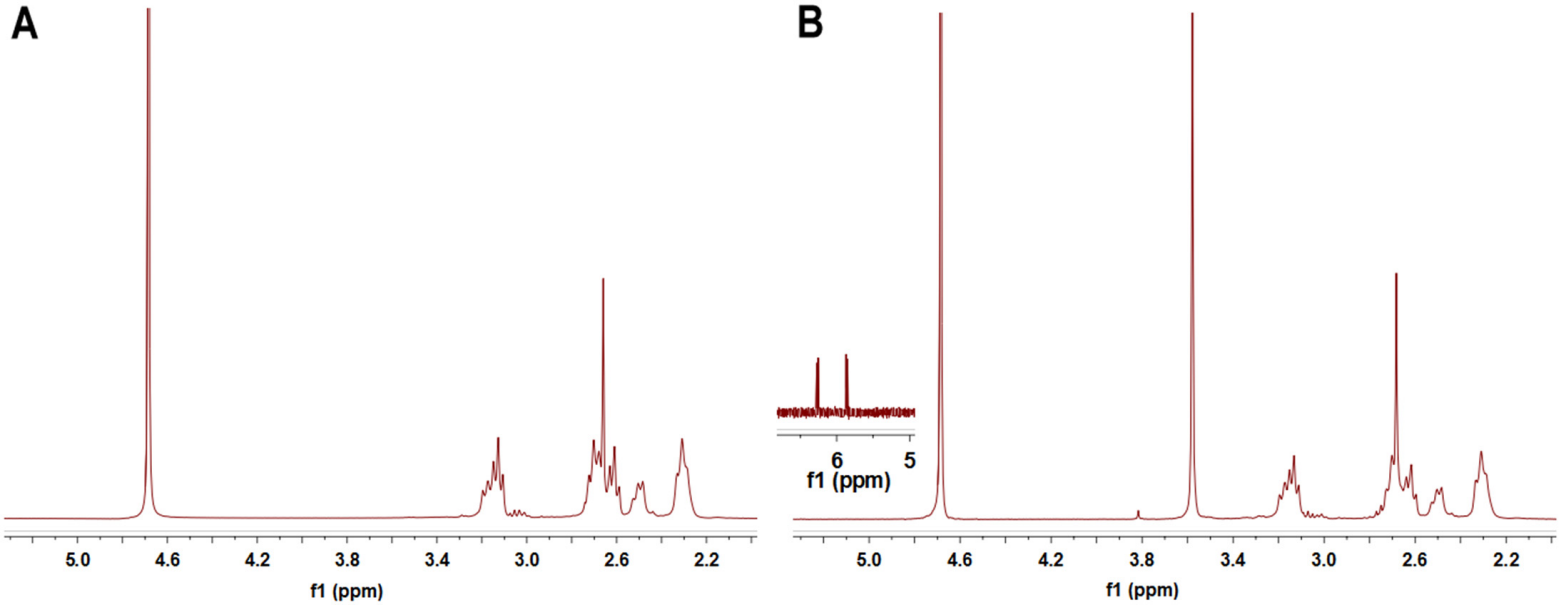
^1^H-NMR of PAMAM (A) and PAMAM-PEG-MAL (B) in D_2_O.

Bifunctional Maleimide polyethylene glycol succinimidyl ester (Mal-PEG-NHS) was adopted to connect the amino group of PAMAM and 5’-thiolated S6 aptamer. Dialysis was used to purify the product. The reaction of PAMAM and Mal-PEG-NHS was monitored by 1H-NMR (300 MHz), with specific peaks for PAMAM (2.3, 2.4, 2.7, 3.1ppm) (Fig 1A), PEG (3.6 ppm), MAL(5.8, 6.3 ppm) (Fig 1B). The ^1^H-NMR data confirmed the formation of PAMAM-PEG-MAL. We used the integrated areas of H-NMR peaks to quantify the ratio between PEG chains and PAMAM. With the assumption of 164 methylene protons per PEG and 2032 per PAMAM, we identified that the PAMAM-PEG-MAL had a PAMAM/PEG proton ratio of 0.32, indicating that each PAMAM had an average of 3.9 PEG chains connection.

### Characterization of PAM-Ap/pDNA nanoparticles

**Fig 2:**
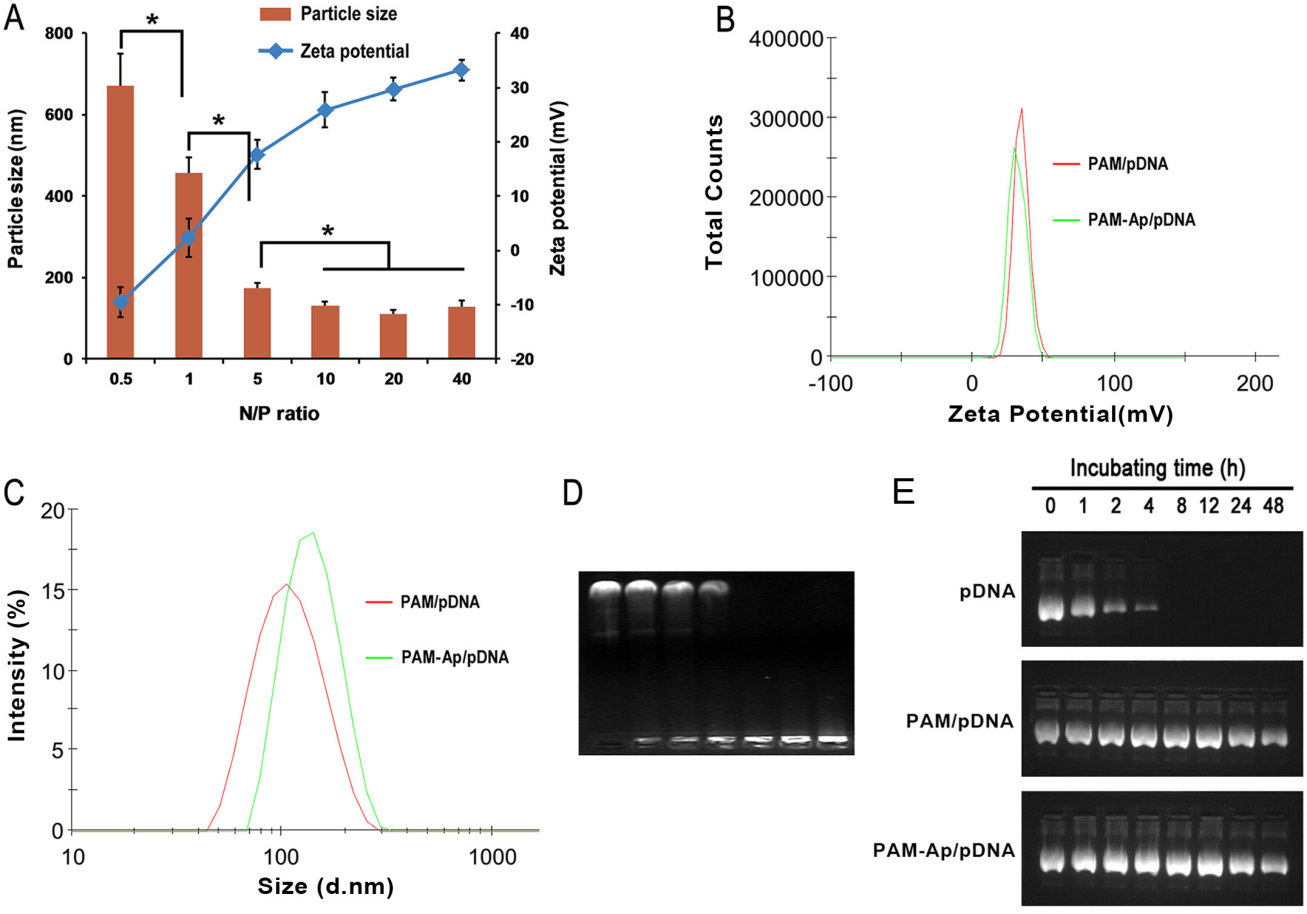
Characterization of PAM-Ap/pDNA NPs. (A) The size and Zeta potential of the PAM-Ap NPs were measured by DLS. (B)The size distribution of PAM/pDNA and PAM-Ap NPs at N/P = 40. (C) The Zeta potential distribution of PAM/pDNA and PAM-Ap NPs at N/P = 40. (D) Gel retardation electrophoresis of PAM-Ap/pDNA NPs. Band 1: Naked pDNA, bands 2–7: PAM-Ap/pDNA NPs were prepared at N/P ratio of 0.5, 1, 2, 4, 8 and 16, respectively. (E) Serum stability test of pDNA, PAM/pDNA and PAM-Ap/pDNA complexes in 50% FBS conditions at 37°C. Data was shown asmean±SD (n = 3).

The freshly prepared PAM-Ap conjugates were dissolved in aqueous conditions and mixed with pDNA to form the nano-scaled particles. The average sizes of PAM-Ap/pDNA NPs range from 100 to 200 nm when the N/P ratio exceeds 5 (Fig 2A). As N/P ratio increased, the size of PAM-Ap/pDNA NPs dropped below 200nm (Fig 2A), and no change in size after that. These results indicated the formation of dense nanoparticles between PAM-Ap and pDNA. As shown in Fig 2B and 2C, the size and zeta potential were slightly changed after the conjugation of PEG and aptamer. The size of PAM-Ap/pDNA further increased to around 150 nm while zeta potential declined to around 31mV, indicating that the PEG and aptamer surrounded the nanoparticle surface. Moreover, PAM-Ap/pDNA NPs had narrower size distribution compared to PAM/pDNA NPs. The pDNA condensation ability of the synthesized PAM-Ap was measured by agarose gel retardation. The gel electrophoresis experiments showed an increased N/P ratio of the PAM-Ap/pDNA NPs (Fig 2D). And at the N/P ratio of 4, complete retardation of PAM-Ap/pDNA was identified.

The stability pDNA in complexes is important for efficient gene delivery. An agarose gel assay was introduced to determine pDNA stability in 50% serum to mimic *in vivo* conditions. The protection effect of complexes against DNA degradation by serum was shown in Fig 2E. The naked pDNA was completely degraded by 50% FBS after 8 h, whereas PAM/pDNA and PAM-Ap/pDNA exhibited protective effects against serum for 48 h (Fig 2E). These results suggested that PAM-Ap could not only bind pDNA, but also protect it from degradation in serum, which could contribute to the potential application of the gene delivery system.

### Cellular uptake of nanoparticles

**Fig 3:**
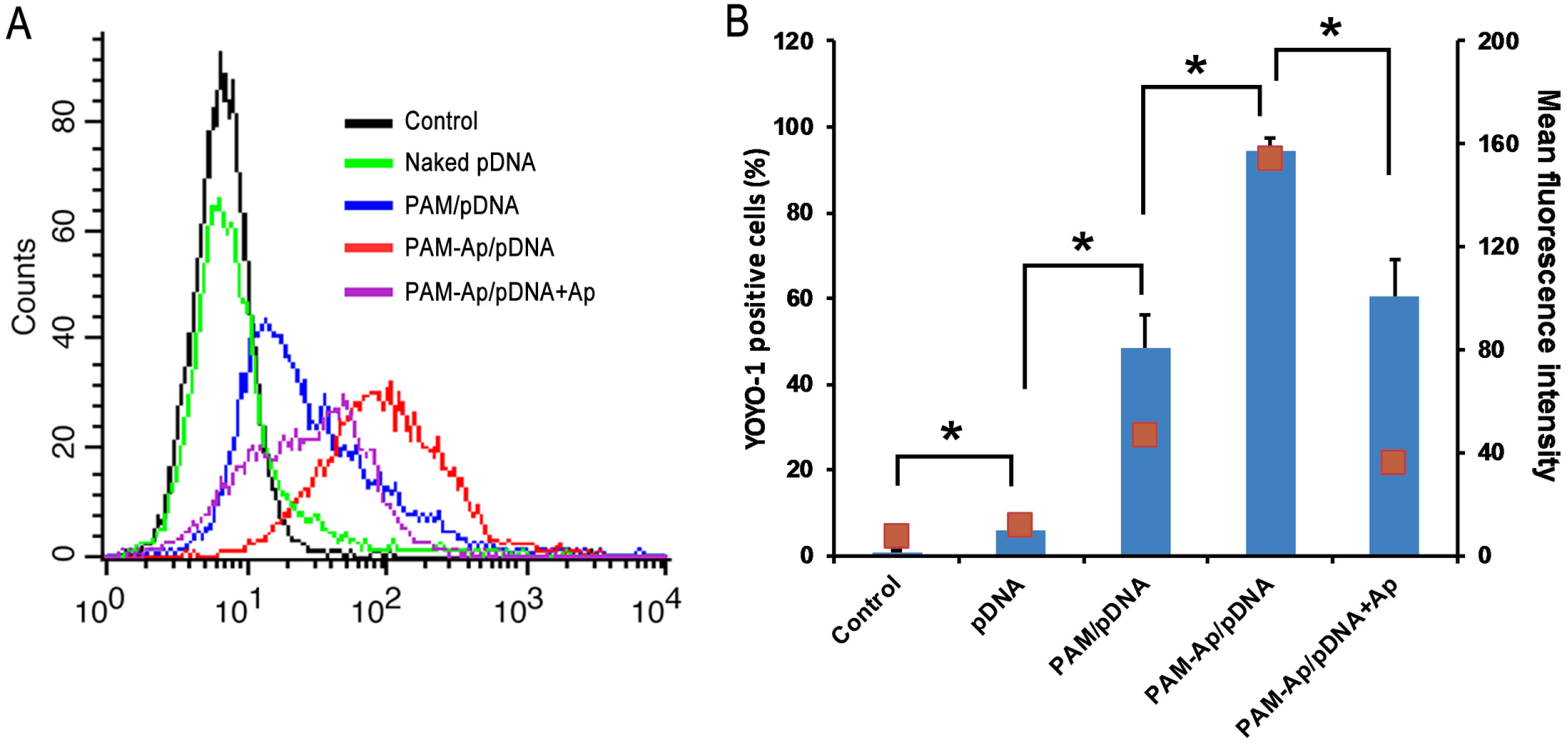
Cellular uptake of YOYO-1 labeled PAM-Ap/pDNA NPs (A) and proportion of YOYO-1 positive cells (B). The untreated cells were used as control. Data was shown as mean±SD (n = 3). *p < 0.05, significant difference between these groups.

The performance of non-viral gene delivery vectors is closely related to the levels of cellular uptake [25]. The green fluorescence emitted from pDNA/YOYO-1 was analyzed to evaluate the cellular uptake of PAM-Ap/pDNA NPs. A549 cells were incubated with naked pDNA, PAM/pDNA or PAM-Ap/pDNA NPs, respectively, and then the YOYO-1 positive cells were quantified by flow cytometry representing the degree of cellular uptake [26]. The proportion of YOYO-1 positive cells increased with N/P ratio, indicating the cellular uptake of NPs was subjected to the N/P ratio to a great extent (Fig 3A and 3B). PAM-Ap/pDNA NPs exhibited higher cellular uptake than nontargeted PAM/pDNA NPs. When treated with PAM-Ap/pDNA NPs at N/P ratio 40, A549 cells exhibited remarkably higher fluorescence signals (94.2% YOYO-1 positive cells) compared with PAM/pDNA NPs treated cells (48.5% YOYO-1 positive cells). These results suggest that the PAM-Ap/pDNA NPs preferentially bind to A549 cells. To further prove that the S6 receptor-mediated uptake of PAM-Ap/pDNA NPs in the cells, we carried out competition experiment by pretreating A549 cells with excess amount of S6 aptamer and revealed an significant lower uptake indicating the free aptamer blocked the binding sites of S6 on cytolemma surface of A549 cells, which suppressed the targeting capability of PAM-Ap/pDNA NPs.

**Fig 4:**
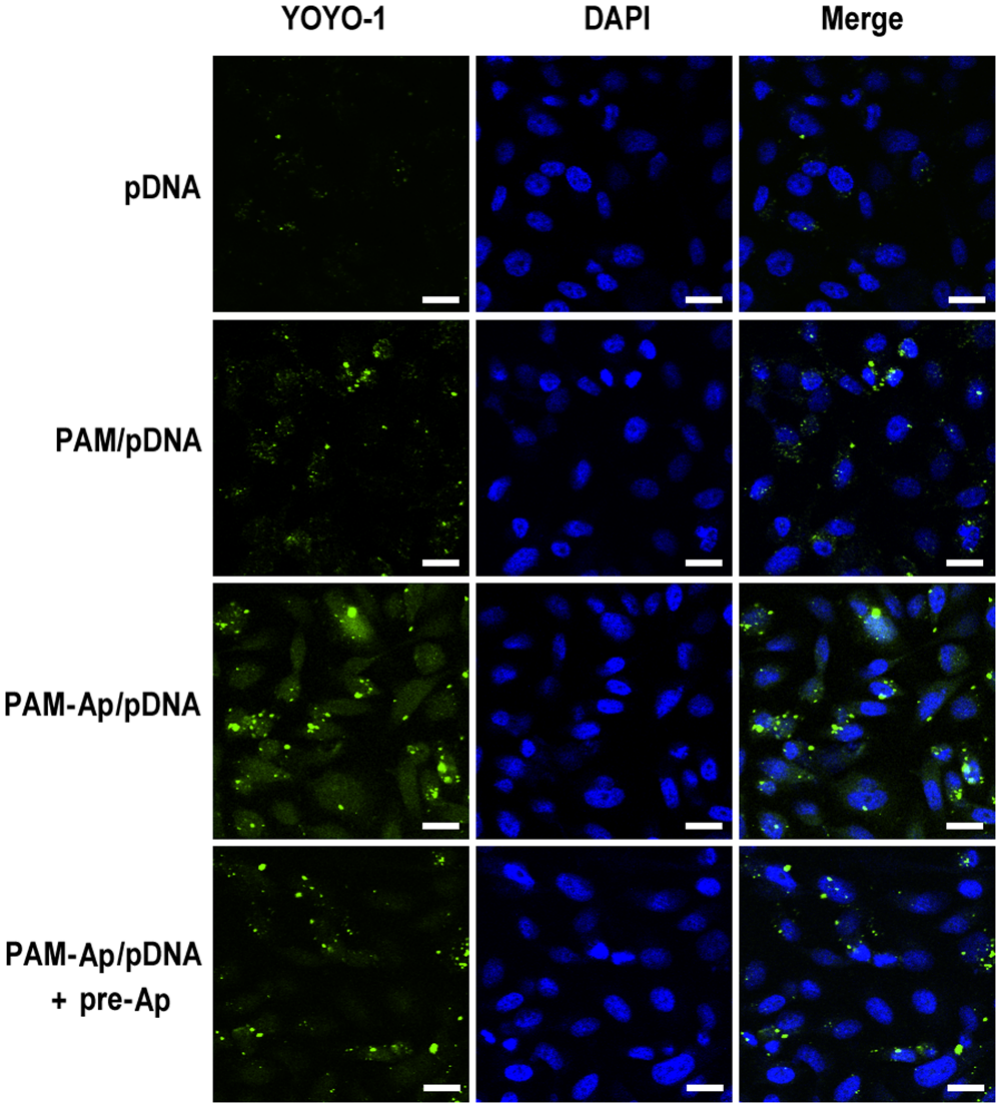
Representative Confocal microscopy images show the cellular uptake of PAM-Ap/pDNA NPs. A549 cells treated with YOYO-1 labeled pDNA complexed with PAM and PAM-Ap (N/P ratio: 40) with or without S6 aptamer pretreated. The cells were incubated at 37°C. All scale bars are 20 μm.

The CLSM experiments were next carried out to further confirm the cellular internalization of receptor-mediated targeted delivery of PAM-Ap/pDNA NPs and distribution of pDNA in A549 cells because CLSM track YOYO-1 labeled pDNA (green fluorescence) formulated in nanocomplexes. Compared to non-targeted PAM/pDNA NPs, A549 cells treated with PAM-Ap/pDNA NPs exhibited stronger green fluorescence signals after 4h incubation, indicating higher cellular uptake of the targeted NPs (Fig 4). Notebaly, fluorescence signal was significantly reduced in the aptamer pretreated A549 cells under similar conditions, suggesting that S6 receptor mediated endocytosis was a major pathway for PAM-Ap/pDNA NPs internalization. Furthermore, most YOYO-1 signals were observed in the cytoplasm, implying the proton sponge effects of PAMAM played a critical role in the process of endosomal/lysosomal escape [27]. In conclusion, our data suggest that PAM-Ap/pDNA NPs show both good cell targeting and endosomal/lysosomal escape properties.

### Transfection efficiency of nanoparticles

**Fig 5:**
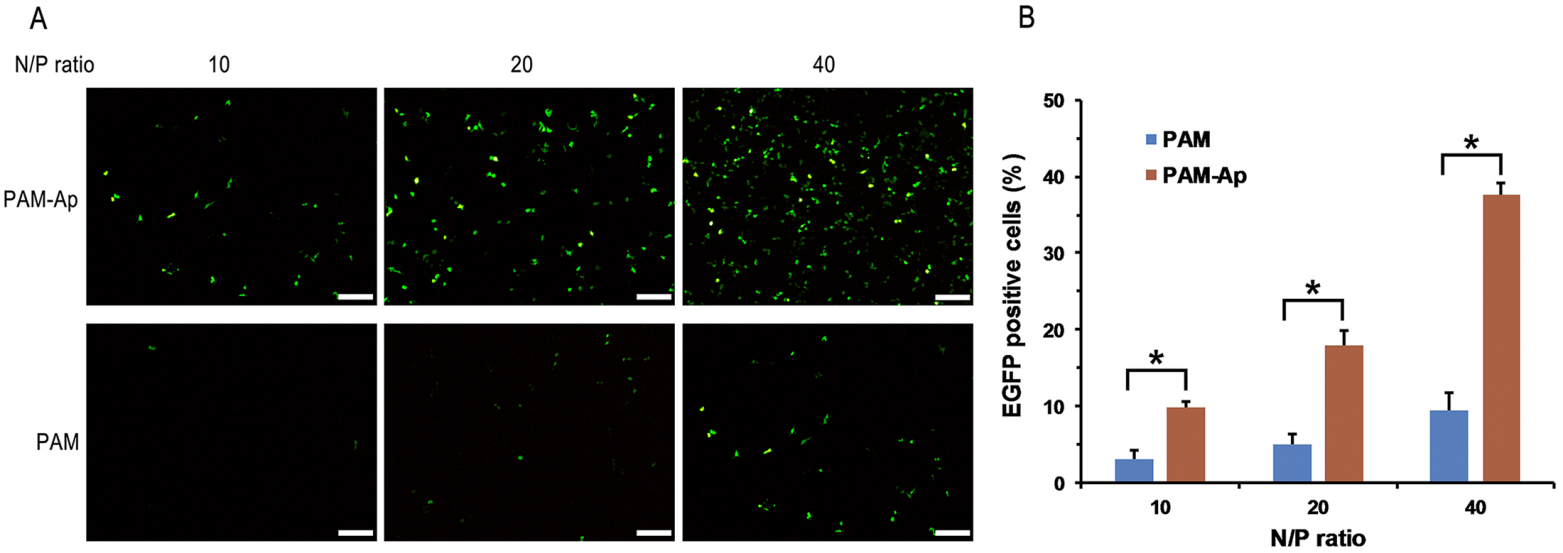
The fluorescent images (A) and quantitative measurements (B) of *in vitro* transfection of PAM /pMiR-34a and PAM-Ap/pMiR-34a NPs on A549 cells at different N/P ratios. Data was shown as mean±SD (n = 3). All scale bars are 200 μm. *p < 0.05, significant difference between these groups.

After demonstrating the efficient cellular uptake of PAM-Ap/pDNA NPs, we next measured transfection efficiency of PAM-Ap/pMiR-34a NPs on A549 cells with a EGFP reporter. Because the pMiR-34a expression vector contains enhanced green fluorescent (EGFP) gene whose expression is driven by the same promoter, the EGFP expression can directly reflect the levels of pMiR-34a expression and can be used to assess transfection efficiency. As shown in Fig 5A and 5B, the gene transfection efficiency of PAM-Ap/pMiR-34a NPs was much higher than that of PAM/ pMiR-34a NPs at different N/P ratios. The exogenous EGFP gene expression increased with the N/P ratio for both nanoparticles. The transfection efficiency was low at N/P ratio of 10 but increased at N/P ratio of 20 and 40. The EGFP expression of PAM-Ap/pMiR-34a NPs on A549 cells was distinctly observed at the N/P ratio of 40, which indicated that nanoparticles achieved effective gene expression efficiency. Similarly, the quantitative measurements of EGFP positive cells confirmed that 37.9% of cells were successfully transfected with PAM-Ap/pMiR-34a NPs when the N/P ratio was 40, whereas it was only 9.5% for the PAM/ pMiR-34a NPs group. Furthermore, there were significant differences of the percentage of transfected cells between the two groups at all N/P ratios. These results suggest that the aptamer conjugation significantly improve the transfection efficiency of PAM-Ap/pDNA NPs. Since the toxicity also increases with the amount of cationic polymers [28, 29], we chose nanoparticles at N/P ratio 40 for the following experiments.

### Influence of gene expression

**Fig 6:**
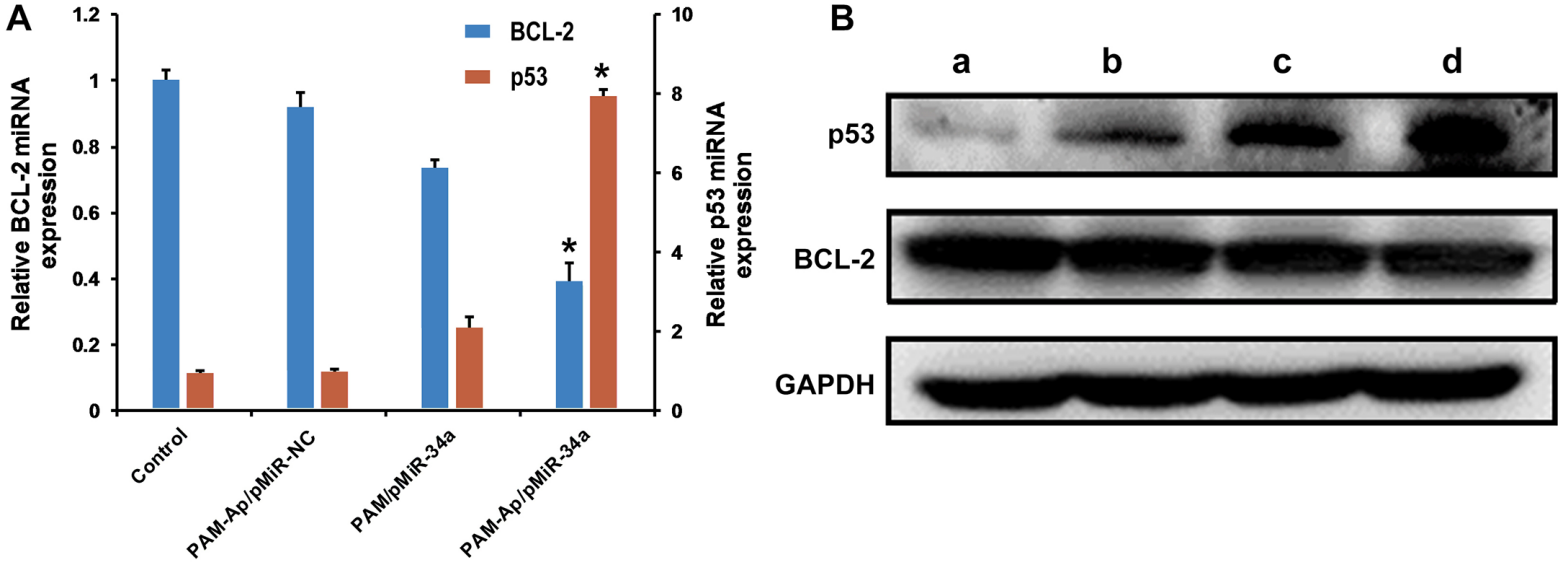
BCL-2 and p53 mRNA (A) and protein (B) expression in the A549 cells. Cells were cultured for 48 h after transfected with PAM-Ap/pMiR-NC NPs, PAM/pMiR-34a NPs and PAM-Ap/pMiR-34a NPs for 4 h. Quantitative real-time PCR analysis was adopted to quantify the mRNA expression, and western blotting was used to determine the protein expression. The untreated cells were used as control. Data was shown as mean±SD (n = 3). *p < 0.05, significant difference as compared with PAM/pMiR-34a.

Over-expression of anti-apoptotic genes in cancer cells is considered as one of the mechanisms for tumor progression. As a potent regulator of apoptosis, BCL-2 has been identified in several types of cancers [30]. BCL-2 has been found to be frequently overexpressed in lung cancer and its anti-apoptotic role is tightly associated with its expression levels [31, 32]. The over-expression of BCL-2 result in cancer cells resistance to chemotherapy drug-induced apoptosis [33]. BCL-2 has been previously identified as a promising downstream target of miR-34a [34]. As a tumor suppressor, the p53 inactivation in response to some cancer associated stress signals is one common genetic alteration in a majority of cancer types [35]. Activated p53 elicits numerous biological outcomes, such as repair of minor damages, regulation of cell-cycle, induction of replicative senescence and apoptosis. The interaction between p53 and miR-34a has been clarified in many types of cancers, including NSCLC [36]. To evaluate the BCL-2 gene suppression and p53 gene activation activity of pMiR-34a transferred A549 cells, we treated A549 cells with PAM-Ap/pMiR-34a, PAM/pMiR-34a or PAM-Ap/pMiR-NC NPs and measured the levels of BCL-2 and p53 mRNAs. The results from qPCR showed that the level of BCL-2 mRNA was significantly down-regulated and p53 mRNA significantly up-regulated in A549 cells (Fig 6A). After 48 h incubation, we observed 60.8% decrease of BCL-2 mRNA expression for PAM-Ap/pMiR-34a, but only 26.5% decrease for PAM /pMiR-34a. Meanwhile, the PAM-Ap/pMiR-34a transfected A549 cells resulted in a 7.93 fold increase of p53 mRNA, significantly higher than that of PAM/pMiR-34a (2.1 fold). Similarly, the expression level of p53 and BCL-2 proteins on the A549 cells were measured by Western blotting (Fig 6B). As expected, the proteins showed the same tendency as the mRNA expression level since p53 protein expression was unregulated and BCL-2 protein was decreased in the PAM-Ap/pMiR-34a transfected A549 cells. Taken together, these results indicate that PAM-Ap/pMiR-34a NPs are capable of influencing related genes and proteins expression in A549 cells.

### Cancer cell growth inhibition

**Fig 7:**
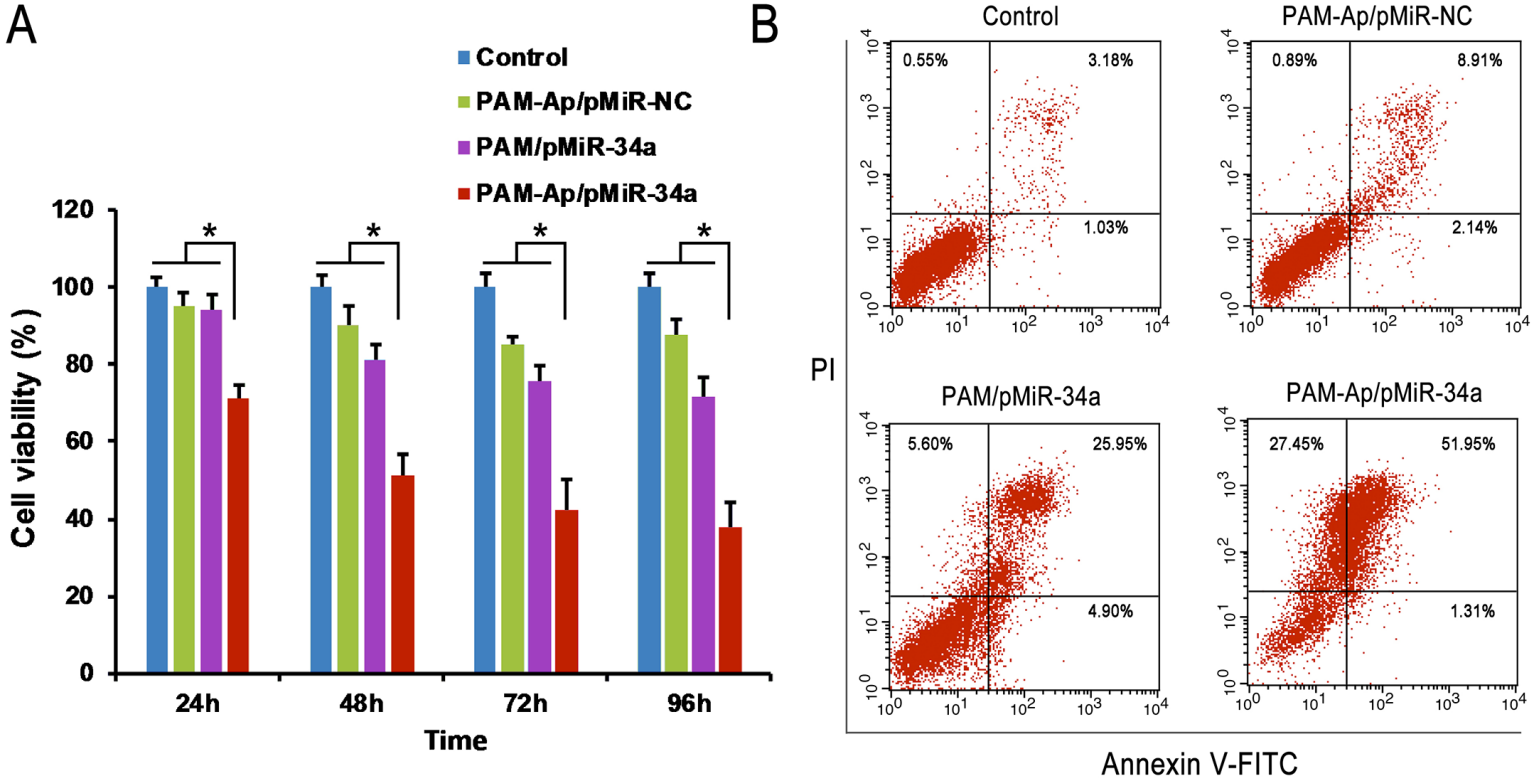
*In vitro* cytotoxicity and apoptosis assay of A549 cell, with the untreated cells as control. (A) In vitro cytotoxicity of PAM-Ap/pMiR-NC, PAM/pMiR-34a and PAM-Ap/pMiR-34a NPs in A549 cells at different time point after transfection. (B) Cellular apoptosis of A549 cells after treatment with PAM-Ap/pMiR-NC, PAM/pMiR-34a and PAM-Ap/pMiR-34a NPs for 48 h. CCK-8 assay was used to evaluate cell viability and flow cytometry was used to determine cell apoptosis with Annexin/PI staining. Data was shown as mean±SD (n = 3). *p < 0.05, significant difference between these groups.

Next, we evaluated the *in vitro* antiproliferation effect of PAM-Ap/pMiR-34a NPs on A549 cells using CCK-8 assay. After 24h, 48h, 72 h or 96h transfection, inhibition rates of cell growth were measured. It was found that PAM-Ap/pMiR-34a NPs exhibited the strongest antiproliferation effects on A549 cells (Fig 7A). Cell viability of PAM-Ap/pMiR-34a NPs treated A549 cells showed 29.8%, 49.7%, 57.5% and 62.4% less than that of control group after 24h, 48h, 72h and 96h of transfection, respectively. As incubation time went on, the gap of cell viability between PAM-Ap/pMiR-34a and other groups increased, and this may be due to the hysteresis of exogenous gene expressed by pMiR-34a. It is worthy to note that PAM-Ap/pMiR-NC NPs slightly inhibited A549 cell growth. Thus, the aptamer also contributed to the antiproliferation effect of PAM-Ap/pMiR-34a. These experimental results indicated that the increase of gene transfection in A549 cells affected cell growth. Thus, PAM-Ap/pMiR-34a NPs has a potential to be a targeted gene delivery system in treating lung cancer.

Apoptosis has generally been considered to be one of the predominant mechanisms for cell death [37, 38]. As shown in Fig 7B, the apoptotic and necrotic cells in the control group were not obvious (<10%). The treatment of PAM/pMiR-34a and PAM-Ap/pMiR-34a NPs significantly increased the percentage of early and late apoptotic cells. Moreover, cells treated with PAM-Ap/pMiR-34a NPs showed the highest percentage of total damaged cells, which was significantly higher than that of the other groups (P < 0.05). The results demonstrated that PAM-Ap/pMiR-34a NPs were more effective in inducing apoptosis of A549 cells than non-targeted PAM /pMiR-34a.

### *In vitro* migration and invasion

**Fig 8:**
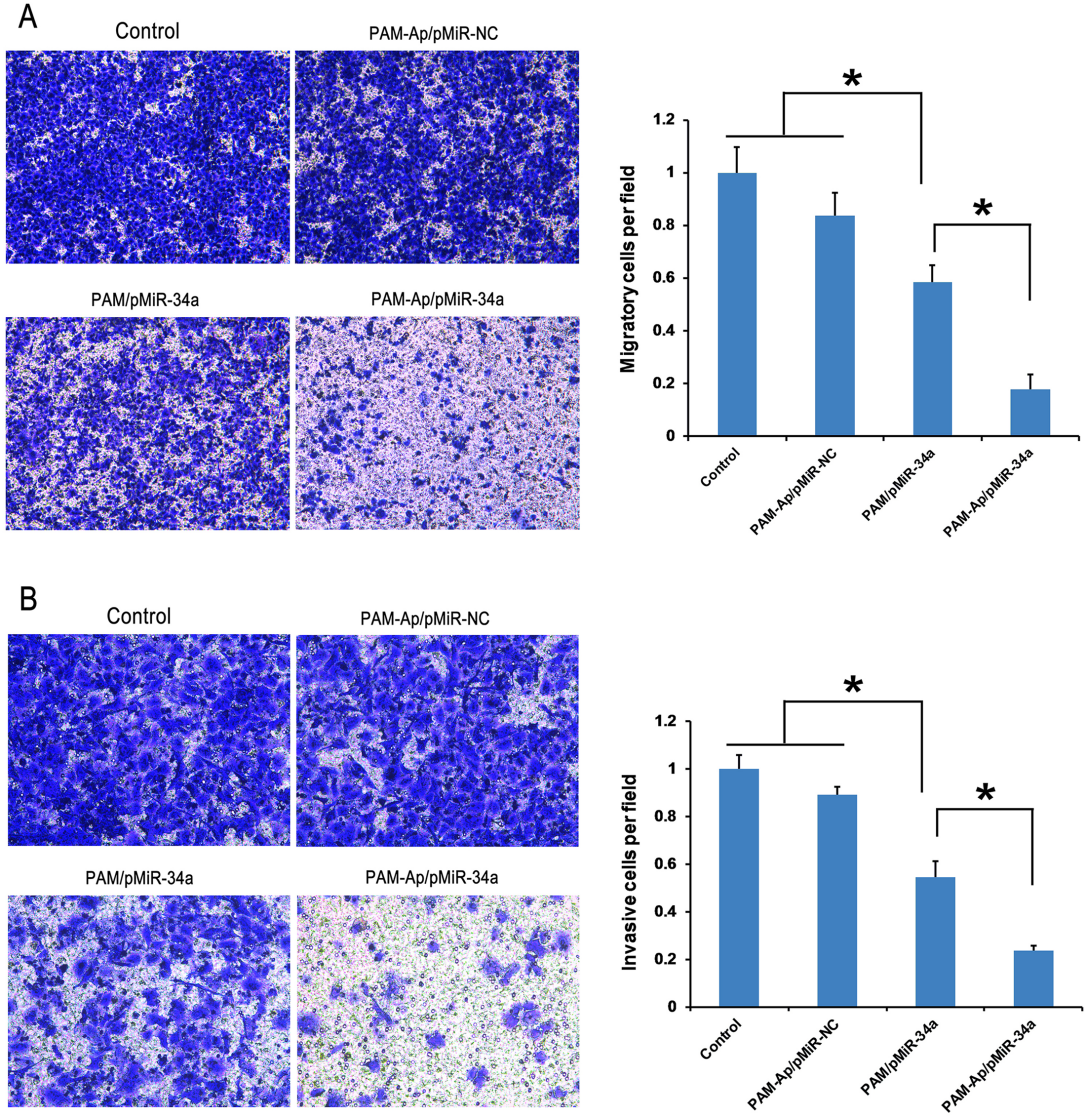
Migration and invasion assay of A549 cells. Cells were pre-treated with PAM-Ap/pMiR-NC, PAM/pMiR-34a and PAM-Ap/pMiR-34a NPs. The untreated cells were used as control. (A) Representative microscopy images of migration and quantitative analysis of migratory cells. (B) Representative microscopy images of invasion and quantitative analysis of invasive cells. Data was shown as mean±SD (n = 3). *p < 0.05, significant difference between these groups.

There are various changes in cancer cells and microenvironment of tissue, and the changes cause cells to migrate or invade into healthy tissues, which results in metastasis of cancer [39]. Therefore, the migration and *in vitro* invasion assays are essential to evaluate the tendency of metastasis process. After transfected with PAM-Ap/pMiR-34a NPs, the migratory and invasive ability of A549 cells were investigated. As shown in Fig 8A and 8B, we found that PAM-Ap/pMiR-34a NPs significantly inhibited the migration and invasion of A549 cells. Remarkably, the migratory and invasive rates of PAM-Ap/pMiR-34a treated cells were 17.7% and 23.8% compared with untreated cells, respectively. However, there was a very minimal impact on the migration and invasion of cells treated with PAM-Ap/pMiR-NC compared with those of the control group, which could be inferred that the miR-34a expression mainly influenced cell migration and invasion rather than S6 aptamer.

## Conclusion

Although the toxicity of dendrimers is one of the factors that limited their clinical applications, dendrimers have been considered as ‘smart’ carriers for their ability as intracellular gene delivery vehicle. PEGylation could decrease toxicity of dendrimers by masking the cationic end groups which has been verified in both *in vitro* and *in vivo* studies [40]. In the present study, We conjugated dendrimer to S6 aptamer through difunctional PEG and demonstrated that PAM-Ap/pMiR-34a NPs has significantly higher cellular uptake and transfection efficiency than non-targeted NPs in NSCLC cell lines. We also investigated the potential antitumor effect of PAM-Ap/pMiR-34a NPs. Our work provides a novel and potential therapeutic solution to deliver microRNA to NSCLC. The systematic evaluation of effectiveness and biocompatibility are important and necessary ahead of the clinical application of this candidate. It is an important venue in our future studies.

## Supporting Information

S1 Table Data of Fig 2A. (XLSX)

S2 Table Data of Fig 3B. (XLSX)

S3 Table Data of Fig 5B. (XLSX)

S4 Table Data of Fig 6A. (XLSX)

S5 Table Data of Fig 7A. (XLSX)

S6 Table Data of Fig 8A and 8B. (XLSX)
